# Effects of playing 1 vs 3 matches in a one-week period on physical performance in young soccer players

**DOI:** 10.5114/biolsport.2022.108700

**Published:** 2021-10-25

**Authors:** Jose Luis Hernández-Davo, Víctor Moreno Pérez, Pedro Moreno Navarro

**Affiliations:** 1 Faculty of Health Sciences, Universidad Isabel I, Burgos, Spain; 2 Sports Research Centre, Miguel Hernandez University of Elche, Alicante, Spain; 3 Center for Translational Research in Physiotherapy, Department of Pathology and Surgery, Miguel Hernandez University of Elche, San Joan, Spain

**Keywords:** Fatigue, Recovery, Soccer, Youth, Performance

## Abstract

The aim of this study was to analyse the effects of playing 1 vs 3 matches in a one-week period on physical performance in young soccer players. Twelve youth soccer players completed a battery of physical tests (countermovement jump [CMJ], 25 m sprint, 5-0-5 agility test, ankle dorsiflexion range of motion [AD ROM]) 72 h after a match. These tests were performed on two different occasions: during a week with 1 competitive match, and during a week in which 3 matches were played. Three matches in a week caused from most likely to very likely impairments in CMJ (ES = 0.81), the 5-0-5 agility test (ES = 1.03), and in AD ROM (ES = 0.46–0.63) compared with the 1 match in a week. For the 25 m sprint test, performance impairments were found in the split times for 10–15 m (ES = 0.72), 15–20 m (ES = 0.52) and 20–25 m (ES = 0.90) compared with 1 match in a week. Jumping, sprinting, change of direction (COD) performance and AD ROM are significantly affected during congested calendars in young soccer players. The monitoring of these variables is a useful tool to assess players’ recovery and may help in determining players’ readiness for the next matches.

## INTRODUCTION

Due to its popularity, soccer has been a topic of great research interest in the last decades [1]. Consequently, several fields such as players’ physical characteristics, game requirements, performance determinants, and fatigue/recovery processes have been extensively studied [2]. Soccer requirements, including a great number of high-intensity actions (e.g., sprints, accelerations, jumps), have been linked to the occurrence of acute fatigue [3]. In addition, the characteristics of the modern competition calendar, with elite players playing more than 70 matches in a season [4], makes the between-matches recovery process difficult. This short period between matches has important implications for injury risk, as several authors have suggested that 3–4 days between matches are insufficient for a proper recovery [4]. Consequently, some authors have found increased injury rates during periods of higher training loads, which are linked to congested calendars [3, 5]. Specifically, higher injury rates (6.2-fold) have been reported in soccer players who played two matches a week compared to those who played only one [5].

Understanding the fatigue induced by a match, as well as the time course of the associated physical impairments, will help in the design of proper strategies to accelerate the recovery process [3]. It seems necessary, therefore, to use sensitive and time-efficient tests to assess the physical impact of a match in the players. Among the different on-field tests employed to assess the fatigue induced by a soccer match, the countermovement jump (CMJ), sprint tests, as well as change of direction (COD) performance, are the most common abilities evaluated. A large amount of research has found physical impairments immediately, 24 h and 48 h after a match [6–8]. Even after a longer (i.e., 72 h) post-match period of time, Silva et al. [2] observed reductions in some physical performance tests. In this last meta-analysis, Silva et al. [2] reported significant physical performance impairments (e.g., CMJ, hamstring strength) 72 h after a match, although other performance variables (e.g., sprint) were affected 24 h after a match, but recovered 72 h after a match. Thus, it seems that different performance capabilities (e.g., sprinting vs jumping) present distinct recovery periods [9].

It should be highlighted that, although congestion in the competitive calendar has been previously linked to increased injury rates, there is a lack of research concerning how physical performance recovery is affected by the number of matches played during a week. In addition, this scarcity of research is even greater in young soccer players. In fact, to the best of the authors’ knowledge, only a few studies [10–12] have analysed match-induced performance impairments in young soccer players. In addition, these articles provide little information about the most common physical performance variables. Specifically, Paul et al. [11] reported data of hip strength values, while de Hoyo et al. [10] and Romagnoli et al. [12] only provide data of CMJ performance. Therefore, the aim of the present study was to analyse how physical performance (i.e., sprinting, jumping, range of motion [ROM]) is affected by playing 1 vs 3 matches in a one-week period in U18 soccer players.

## MATERIALS AND METHODS

### Participants

Twelve young male soccer players (age = 16.9 ± 0.6 years; height = 174.3 ± 5.3 cm; body mass = 65.1 ± 8.0 kg) from two different teams voluntarily participated in the study. At the time of the study, the subjects from both teams were competing in the same regional league. All subjects reported at least 10 years of experience in soccer training. Their habitual training consists of 3 x 90-minute sessions per week, and normally, 1 match during the weekend. The habitual microcycle includes a recovery-oriented session (session 1), a conditioning-oriented session (session 2) and a tactical/tapering-oriented session (session 3). All subjects and their guardians were fully informed about the study procedures, and signed a written informed consent form approved by an Ethics Committee, in accordance with the recommendations of the Declaration of Helsinki.

### Design

**FIG. 1:**
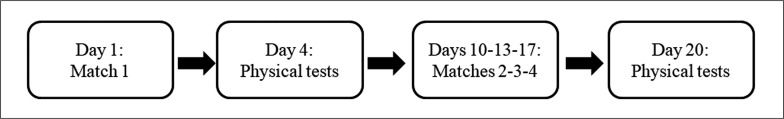
Schedule of the study procedures.

The study began after a complete one-week period of habitual training (i.e. 7 days without a match). The study was carried out in a 20-day period in which the subjects played 4 matches and attended two testing sessions. The first testing session was performed 72 h after the first match. The second testing session was performed 72 h after the fourth match (matches 2, 3 and 4 were separated by 72 and 96 h respectively). Figure 1 shows the schedule of the study. The number of minutes played was recorded during all matches. During the testing sessions, all players performed the following tests: CMJ, 25 m sprint, 5-0-5 agility test, and ankle dorsiflexion range of motion (AD ROM). All subjects included in the data analysis played at least 70 minutes per match.

### Methodology

Before testing, all subjects completed a standardized warm-up consisting of 5 minutes of self-selected light-to-moderate runs, lower-limb dynamic stretching and sub-maximal attempts of sprinting and jumping tests.

Jumping ability was assessed by measuring a CMJ with an electronic contact mat (Tapeswitch Signal Mat, Tapeswitch Corporation America, New York, USA). Electronic contact mats have previously been shown to be valid and reliable tools for jumping assessment (Rago et al., 2018; Tenelsen et al., 2019). CMJ height showed high intraclass correlation coefficient (ICC) values (0.87; CI = 0.75–0.93) and low standard error of measurement (4.87%; CI = 3.90–6.56%). Jumps were performed with a self-selected depth, with the hands on the hips and with the instruction to jump as high as possible. Each participant performed two attempts separated by 2 minutes of passive recovery, using the best trial for statistical analysis.

The time during a 25 m sprint in a straight line was measured by means of photocells (Witty System, Microgate, Bolzano, Italy). In addition, a photocell was placed every 5 m to record the split times. The players performed two maximal sprints interspersed with 2 minutes of passive recovery. Each sprint was initiated from an individually chosen standing position 50 cm behind the photocell. The fastest attempt was used for analysis.

The ability of the players to perform a single, rapid 180° COD over a 5 m distance was measured (photocells) using a modified version (stationary start) of the 5-0-5 agility test [7]. Players started in a standing position with their preferred foot behind the starting line, followed by accelerating forward at maximal effort. Each player performed one trial pivoting on both right and left feet, separated by 2 minutes of passive recovery. The fastest trial was used for analysis.

AD ROM was evaluated using the knee-to-wall method. The AD ROM was quantified with a bi-level inclinometer (ISOMED, Portland, OR, USA) positioned on the midline of the tibia, 15 cm proximal to the distal tip of the lateral malleolus [13]. Players were required to lunge the knee forward until maximum AD was reached, which was identified as the heel lifting off the ground [14].

### Statistical Analysis

All data were analysed for practical significance by means of magnitude-based inferences [15] using the spreadsheets available at www.sportsci.org. The chances that any physical performance value was greater/similar/smaller between testing sessions and/or between groups were subsequently calculated (standardized difference and 90% confidence level [CL]). Qualitative mechanistic assessment of the magnitude of change was also included. If the 90% CL overlapped small positive and negative values, the magnitude of the changes was termed unclear; otherwise it was deemed to constitute the observed magnitude. The effect size (ES) values were categorized as trivial (< 0.2), small (0.2–0.5), moderate (0.5–0.8) and large (> 0.8).

## RESULTS

The differences in the physical variables measured in the two periods of the study are shown in Table 1. The CMJ (large ES), 5-0-5 agility test (large ES), and AK ROM with both dominant (moderate ES) and non-dominant (small ES) legs were from *likely* to *most likely* affected when comparing the 3-match period with the 1-match period. The 25 m sprint test showed *unclear* effects, but a moderate magnitude of change (ES = 0.68).

**TABLE 1:** Difference in physical tests between the 1 match and the 3 matches period.

Variable	1 match period	3 matches period	ES (90% CL)	Chances	Qualitative assessment
**CMJ (cm)**	39.94 ± 4.49	36.05 ± 4.83	–0.81 (–1.15, –0.47)	0/0/100	Most likely lower
**Sprint 25 m (s)**	3.61 ± 0.07	3.66 ± 0.14	0.68 (–0.46, 1.82)	77/14/10	Unclear
**5–0–5 (s)**	2.68 ± 0.09	2.79 ± 0.10	1.03 (0.44, 1.62)	99/1/0	Very likely greater
**ROM AD DOM (°)**	41.67 ± 7.18	36.83 ± 6.94	–0.63 (–0.90, –0.36)	0/1/99	Very likely lower
**ROM AD NDOM (°)**	40.33 ± 6.43	36.75 ± 7.90	–0.46 (–0.71, –0.22)	0/4/96	Very likely lower

Note: CMJ = countermovement jump; DOM = dominant; NDOM = non-dominant; AD = ankle dorsiflexion; ROM = range of movement.

**FIG. 2:**
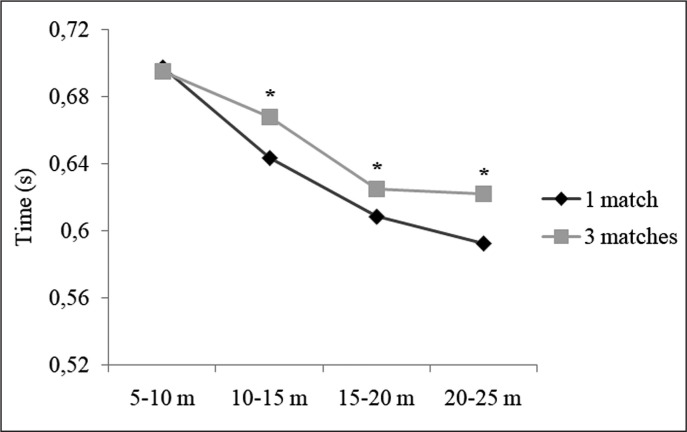
Comparison of split times during the 25 m sprint test between the 1-match and the 3-match period. Note: * = significantly different to 1 match in a week.

After dividing the 25 m sprint into 5 m split times, *likely* to *very likely* greater values (moderate to large ES) were found for the split times 10–15 m, 15–20 m and 20–25 m in the 3-match period (Table 2 and Figure 2).

**TABLE 2:** Time spent within the different sections of the 25 m sprint between the 1 match and the 3 matches period.

Variable	1 match period	3 matches period	ES (90% CL)	Chances	Qualitative assessment
**5–10 m (s)**	0.697 ± 0.032	0.695 ± 0.041	–0.06 (–1.00, 0.89)	32/29/39	Unclear
**10–15 m (s)**	0.643 ± 0.036	0.668 ± 0.029	0.72 (–0.08, 1.53)	87/10/3	Likely greater
**15–20 m (s)**	0.608 ± 0.031	0.625 ± 0.024	0.52 (–0.20, 1.23)	78/17/5	Likely greater
**20–25 m (s)**	0.592 ± 0.028	0.622 ± 0.028	0.90 (0.41, 1.40)	99/1/0	Very likely greater

## DISCUSSION

The aim of this study was to compare the effect of playing 1 vs 3 matches in a week on physical performance variables (CMJ, sprint, COD, ROM) in young soccer players. The main finding of the present study was that, after playing 3 matches in a week, soccer players presented moderate to large impairments in jumping performance as well as in the COD test. In addition, other physical variables such as AD ROM and some split times in the sprint test were also impaired 72 h after the match when comparing the 3- vs 1-match period.

The CMJ is one of the most common tests used to evaluate the lower-limb muscles’ explosiveness. Several studies have previously reported decreases in CMJ performance even after a long period (e.g., 48–72 h) since the soccer match [3, 12, 16]. The results of the present study expand these previous data, as impairments in CMJ were observed 72 h after the 3-match period compared with the 1-match period (ES = -0.81). Previous research has shown increases in concentrations of some substances (e.g., creatine-kinase, urea, uric acid) after a soccer match [3, 9, 10, 16]. Although not measured in this study, the potential mechanisms explaining the CMJ impairments may be linked to disruptions within the muscular fibres [3]. Thus, myofiber disruptions have been suggested to be responsible for the decline in the force-generation capacity after a soccer match [3, 17]. It seems, therefore, that playing 3 matches in a one-week period causes a great impact on the lower-limb muscles, which are not completely recovered 72 h after the last match.

Straight sprinting is the most common action before goal situations [18], highlighting the importance of players’ speed in soccer success. In the present study, the players showed a performance impairment in the sprint test when comparing the evaluation after the 3-match period compared with the 1-match period (Table 2 and Figure 2). Interestingly, these impairments were greater in the split times more linked to maximal velocity (i.e., 15–20 m, and 20–25 m). As discussed above, high-intensity actions within the soccer game results in muscle damage (especially in type II fibres), and this muscle damage has been linked to sprinting performance impairments [9]. In a recent meta-analysis, Silva et al. [2] found that straight sprint performance is slightly affected 24 and 48 h after a match, but not affected 72 h after a match. In our study, testing was performed 72 h after a match, but, unlike previous research, the tests were performed after playing 3 matches in a week. This fact highlights that during periods of a congested calendar, sprinting performance of soccer players is not recovered 72 h after a match.

Although COD ability is considered essential for successful participation in most team sports [19], little is known about how a soccer match affects COD performance. It is difficult to compare the data of the present study with previous research, as a wide variety of COD tests have been used: shuttle sprint [20], T test [1], and the L-agility run test [21]. Silva et al. [2] found in their meta-analysis that COD performance was affected 24 h after a match, but unclear effects were found at 48 and 72 h after a match. However, in the present study, large impairments were found when comparing players’ performance 72 h after a match during a 1-match week with a match during a 3-match week. COD actions require rapid transitions from eccentric to concentric muscle actions [22, 23]. Soccer game requirements, with some maximal-sprint as well as numerous deceleration and acceleration actions, have been proposed as mechanisms causing great muscle damage [22]. This muscle damage together with inter-muscular coordination deficiencies may explain the impairments in COD performance after playing 3 matches in a one-week period.

A reduced AD ROM has been linked to potential injury mechanisms such as smaller knee-flexion displacements and greater ground reaction forces during landing tasks [24, 25]. Therefore, monitoring AD ROM could be useful to control the injury risk associated with reductions in this variable. Charlton et al. [26] found reductions in AD ROM immediately after an Australian football match, although the ROM recovered to baseline values 24 h after the match. In another study, Wollin et al. [13] observed smaller, although non-significant, decreases in AD ROM 48–72 h after a soccer match. In the present study, AD ROM was reduced when comparing the value after the 3-match week compared with the 1-match week. It seems, therefore, that although some studies have shown that 48–72 h is enough to recover baseline AD ROM values, this period is insufficient during congested calendars.

The present study has some limitations. The main limitation is the lack of a baseline measure. This baseline value could have been used to ascertain whether the performance in the two testing sessions was different than in a “non-fatigued” state. The absence of GPS measures is another limitation of the study. It should be recorded in future studies to evaluate the potential influence of decrements in isolated testing performance and changes in match-related activities. In addition, the sample size and the characteristics of the players do not permit generalization of the results. Future research is needed to increase the data about how consecutive matches play a role in the time-course recovery of physical performance.

## CONCLUSIONS

The results of the present study are of significant practical use for coaches and athletes. Large performance impairments were found between the tests performed after playing 3 matches in a week compared with the 1-match period. Thus, coaches should know that, after a congestive period of competitions (e.g., 3 matches in 7–10 days), soccer players are not completely recovered 72 h after the last match. This fact is extremely important, as previous research has shown increases in injury rates during congested calendars, and athlete monitoring should be specially performed during these periods. In conclusion, jumping performance, maximal velocity, change of direction performance and ankle dorsiflexion ROM values have been shown to be sensitive to congested calendars. During congested calendars, performance in these variables could be decreased by an insufficient recovery period. This information may be used for decision purposes (e.g., starting players) and also to reduce the incidence of injuries.

## Conflict of interests

The authors report no conflicts of interests concerning the contents of this paper.
